# Semi‐Continuous Versus Continuous Suturing Techniques in Bronchial Anastomosis Following da Vinci Robotic‐Assisted Sleeve Lobectomy

**DOI:** 10.1111/1759-7714.70206

**Published:** 2026-02-02

**Authors:** Zhiqiao Chen, Yongxin Fan, Xinyu Zhu, Shuyuan Li, Qi Tang, Xuanyi Zong, Shoujie Feng, Cheng Zhang, Teng Sun, Yong Ge, Hao Zhang

**Affiliations:** ^1^ Thoracic Surgery Laboratory Xuzhou Medical University Xuzhou China; ^2^ Department of Thoracic Surgery Affiliated Hospital of Xuzhou Medical University Xuzhou China

**Keywords:** bronchial anastomosis, central type carcinoma of lung, continuous suture, semi‐continuous suture

## Abstract

**Background:**

In robot‐assisted thoracoscopic (RATS) bronchial sleeve lobectomy, despite the continuous suturing (CS) technique's widespread adoption, the safety and advantages of the semi‐continuous suturing (SCS) technique remain inconclusive.

**Methods:**

Patients undergoing RATS bronchial sleeve lobectomy for central Non‐Small Cell Lung Cancer (NSCLC) between January 2020 and December 2024 were retrospectively enrolled and stratified into two cohorts based on anastomotic technique: the CS group and the SCS group. Perioperative outcomes were compared between the two groups.

**Results:**

The SCS group (*n* = 18) demonstrated significantly shorter anastomotic time than the CS group (*n* = 14) (median 28 min [24–33] vs. 45 min [32–52]; *p* < 0.001), with a 21‐min reduction in operative time (median 135 min [110–185] vs. 156 min [138–212]; *p* = 0.040). No statistically significant differences were observed in: overall complication rates (anastomosis‐specific: 11.1% vs. 21.4%, *p* = 0.425; systemic: 22.2% vs. 42.9%, *p* = 0.212); 90‐day mortality (0% vs. 7.1%, *p* = 0.467); late stenosis rate (0% vs. 7.1%, *p* = 0.249) or reoperation rate (5.6% vs. 14.3%, *p* = 0.401); postoperative recovery metrics (extubation time and hospital stay, *p* > 0.05).

**Conclusions:**

SCS can safely reduce bronchial anastomosis time in RATS sleeve resection and is recommended as the preferred technique for optimizing operative efficiency.

## Graphical abstract

Semi‐continuous suturing significantly reduces bronchial anastomosis time during robotic sleeve lobectomy compared to continuous suturing, without increasing complication rates, offering a safer and more efficient technical option for bronchial reconstruction.

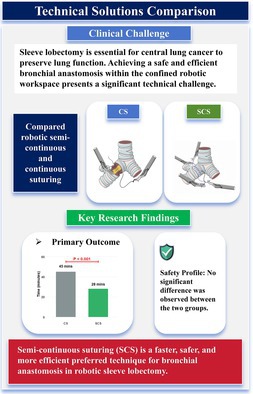

## 1 Introduction

Bronchial sleeve lobectomy has been established as a definitive surgical procedure for central lung tumors [1]. Compared to pneumonectomy, this technique preserves pulmonary parenchyma more effectively, leading to superior postoperative quality of life and pulmonary function, oncological radicality, and significantly reducing postoperative morbidity and mortality [2]. The technical success of this procedure—especially when performed through minimally invasive techniques such as video‐assisted thoracoscopic surgery (VATS) or robot‐assisted thoracoscopic surgery (RATS)—primarily depends on the precision and integrity of bronchial anastomosis [3].

Current bronchial anastomosis techniques commonly employ continuous suturing (CS) and semi‐continuous suturing (SCS) [4]. Bayram et al.'s animal experiment [5] demonstrated that CS significantly shortened anastomotic time compared to interrupted suturing (9.6 min vs. 15.2 min) following sleeve resection, with histological examination confirming equivalent anastomotic healing efficacy between both techniques. However, the traditional CS technique still exhibits inherent limitations when using smooth sutures (e.g., 3–0 Prolene): long‐distance single‐thread suturing requires repeated traction to maintain tension, which easily leads to suture entanglement or loosening, thereby increasing the technical difficulty of fine manipulation in robotic or thoracoscopic procedures [6]. The currently prevalent SCS technique typically utilizes double‐threaded 3–0 Prolene smooth sutures [7]. SCS resolves critical constraints inherent to traditional CS through its double‐threaded segmental technique (separate suturing of membranous and cartilaginous portions) [8]:① Segmented knotting reduces single‐thread length, lowering suture management difficulty; ② Independent tension adjustment prevents local over‐tightening, offering a novel solution to suture management challenges in minimally invasive anastomosis.

Although both CS and SCS techniques have been applied in clinical practice [9], there remains a lack of high‐level evidence comparing the advantages and disadvantages of different suturing techniques (particularly between SCS and traditional CS) in robot‐assisted bronchial sleeve lobectomy. Therefore, this study aims to compare and analyze the application outcomes of CS technique versus SCS technique in RATS bronchial sleeve lobectomy, evaluating their differences in operative time, anastomotic procedural convenience, perioperative complications [10] (particularly anastomotic leakage and stenosis), and short‐term prognosis, thereby providing evidence‐based medical support for optimizing surgical techniques and establishing standardized operative procedures.

## 2 Patients and Methods

### 2.1 Study Design

**FIGURE 1:**
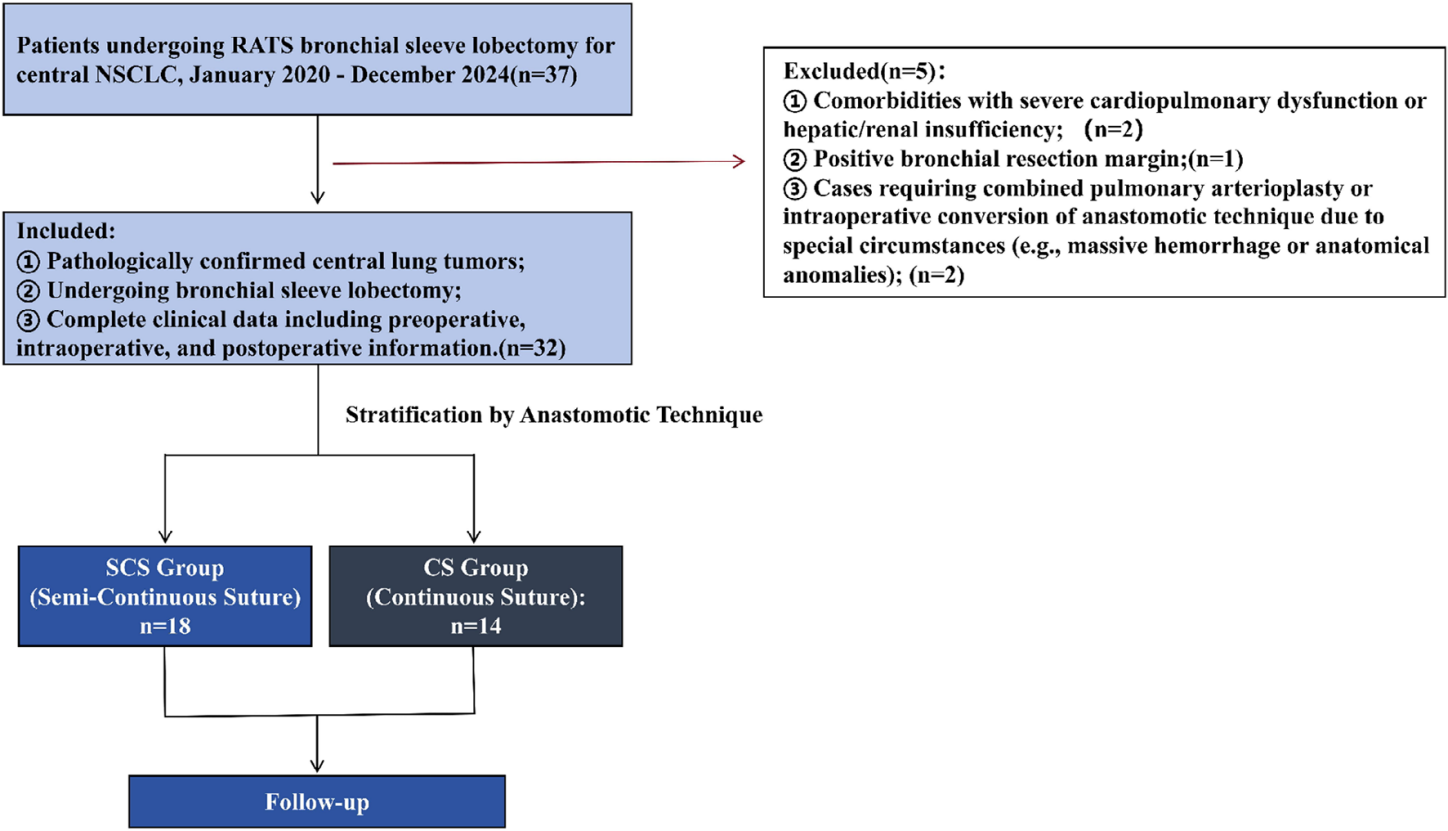
Patient selection flowchart for robotic sleeve lobectomy cohort (*n* = 32 enrolled).

A retrospective cohort study was conducted, enrolling patients with central NSCLC who underwent RATS bronchial sleeve lobectomy between January 2020 and December 2024. Patients were divided into two groups based on anastomotic technique(Figure 1):CS Group: Single‐thread continuous anastomosis throughout (membranous portion + cartilaginous portion) SCS Group: Double‐threaded technique (3–0 Prolene semi‐continuous anastomosis).

### 2.2 Study Population

Inclusion criteria were ① Pathologically confirmed central lung tumors; ② Undergoing bronchial sleeve lobectomy; ③ Complete clinical data including preoperative, intraoperative, and postoperative information.

Exclusion criteria were ① Comorbidities with severe cardiopulmonary dysfunction or hepatic/renal insufficiency; ② Positive bronchial resection margin; ③ Cases requiring combined pulmonary arterioplasty or intraoperative conversion of anastomotic technique due to special circumstances (e.g., massive hemorrhage or anatomical anomalies).

### 2.3 Data Collection

Clinical data were collected through the hospital electronic medical record system, including:

#### 2.3.1 Demographic Data

Age, sex, body mass index (BMI), smoking history, preoperative comorbidities (e.g., hypertension, diabetes), tumor characteristics (clinical stage, pathological type), and preoperative pulmonary function parameters (FEV1, DLCO).

#### 2.3.2 Intraoperative Data

Operative time, anastomotic time, intraoperative blood loss, and number of lymph nodes dissected.

#### 2.3.3 Postoperative Data

Extubation time, postoperative hospital stay, perioperative complications (with emphasis on bronchopleural fistula, anastomotic stricture, pneumonia, and arrhythmia), and 90‐day surgical mortality.

### 2.4 Surgical Technique

All procedures were performed under general anesthesia with double‐lumen endotracheal intubation [11]. Following preparation of the da Vinci robotic system, the positions of the robotic arms were adjusted. Three incisions were made on the affected hemithorax for insertion of the thoracoscope and left/right robotic arms, with an additional auxiliary port created for the assistant's operation. After adhesiolysis and hilar dissection, the pulmonary vein was divided first, followed by division of the interlobar fissure and pulmonary artery according to intraoperative findings. Lymphadenectomy around the affected bronchus was performed to achieve adequate exposure. Upon confirmation of negative resection margins by intraoperative frozen section [12], bronchial anastomosis was performed using either the continuous or SCS technique. Following completion of the bronchial anastomosis, we performed an air leak test to confirm anastomotic integrity. Routine hemostasis was achieved, and a chest tube was placed for drainage. Postoperative management included standard protocols for antibiotic prophylaxis, nebulization, and expectorant therapy.

**FIGURE 2:**
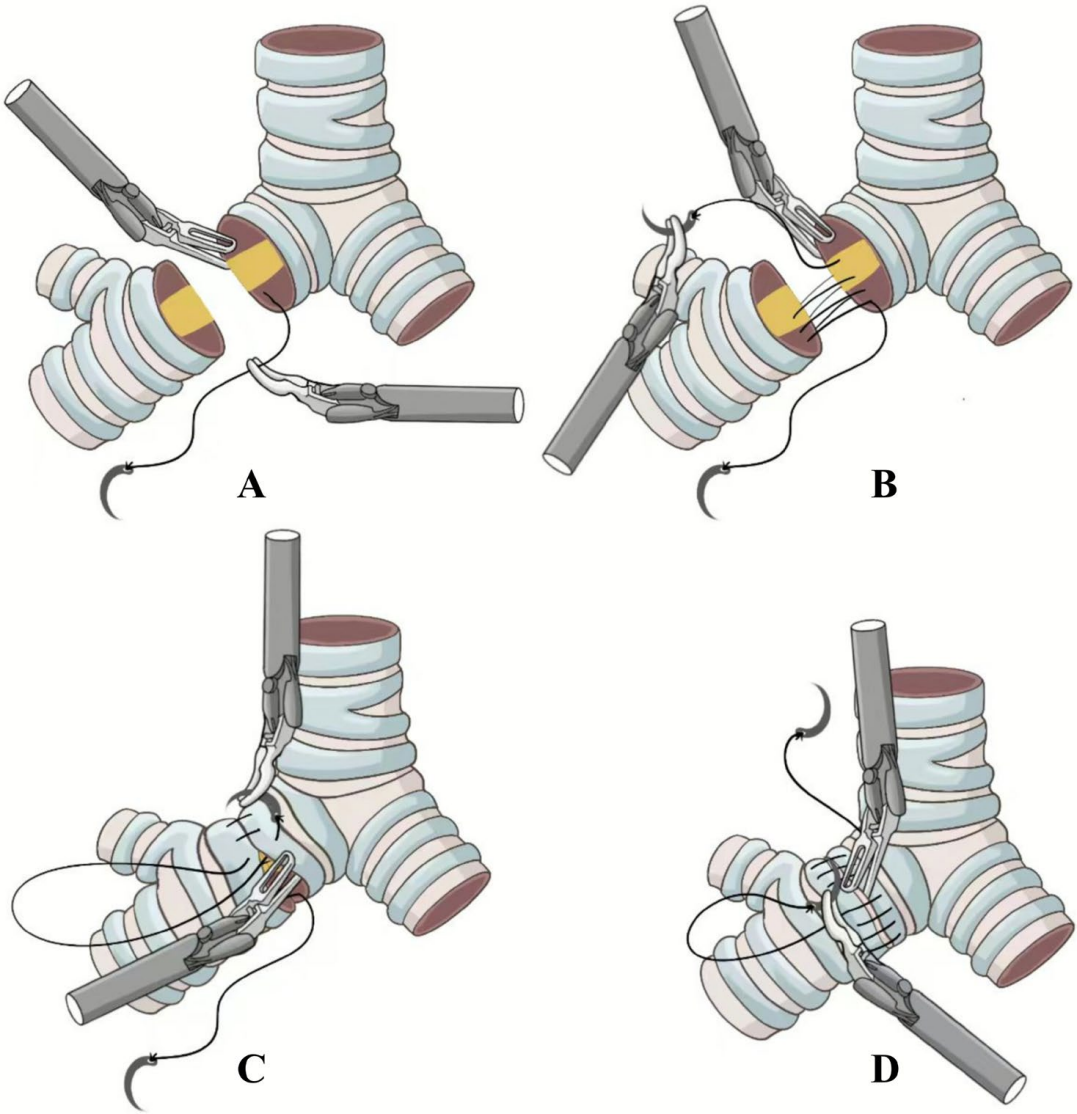
Continuous suture group (CS): A single‐thread continuous anastomosis was performed throughout both the membranous and cartilaginous portions. (A) Initial needle penetration at the cartilaginous portion of the posterior bronchial wall. The needle is introduced submucosally at one resection margin and passed through to the adventitial layer. (B) Full‐thickness suturing across bronchial stumps. The needle is reinserted from the adventitia to the submucosa at the opposing margin, completing transmural approximation. (C) Continuous circumferential suturing with tensioning. Sutures advance along the bronchial wall (0.7‐cm margin, 0.5‐cm interval), tightened bidirectionally every 1–3 stitches. (D) Extracorporeal knot tying at the anterior aspect. Both suture ends meet anteriorly for final knot fixation after endotracheal suctioning.

The anastomosis was performed using 3–0 Prolene sutures, beginning at the cartilaginous portion of the posterior bronchial wall. The needle was introduced submucosally at one resection margin and passed through to the adventitial layer, then reinserted from the adventitia to the submucosa at the opposing margin. After placing four to six stitches, the suture was carefully tightened bidirectionally to ensure precise approximation of the bronchial walls. Using a double‐armed suture technique, both needles were then advanced laterally along the bronchial walls until they met at the anterior aspect, where the knot was tied extracorporeally. Throughout the procedure, a stitch margin of 0.7 cm and interval of 0.5 cm were maintained, with the interval adjusted to 0.6–0.7 cm on the wider lumen side when necessary to accommodate diameter discrepancies. The sutures were tensioned every 1–3 stitches to achieve airtight alignment, with particular attention paid to performing full‐thickness suturing of the membranous wall to prevent layer separation. Prior to final knot tying, endotracheal suctioning was performed to clear secretions and blood from the lumen. Following anastomosis, an air leak test was performed, and if air leakage was identified, additional sutures were placed to reinforce the repair while carefully avoiding damage to the original suture line (Figure 2).

**FIGURE 3:**
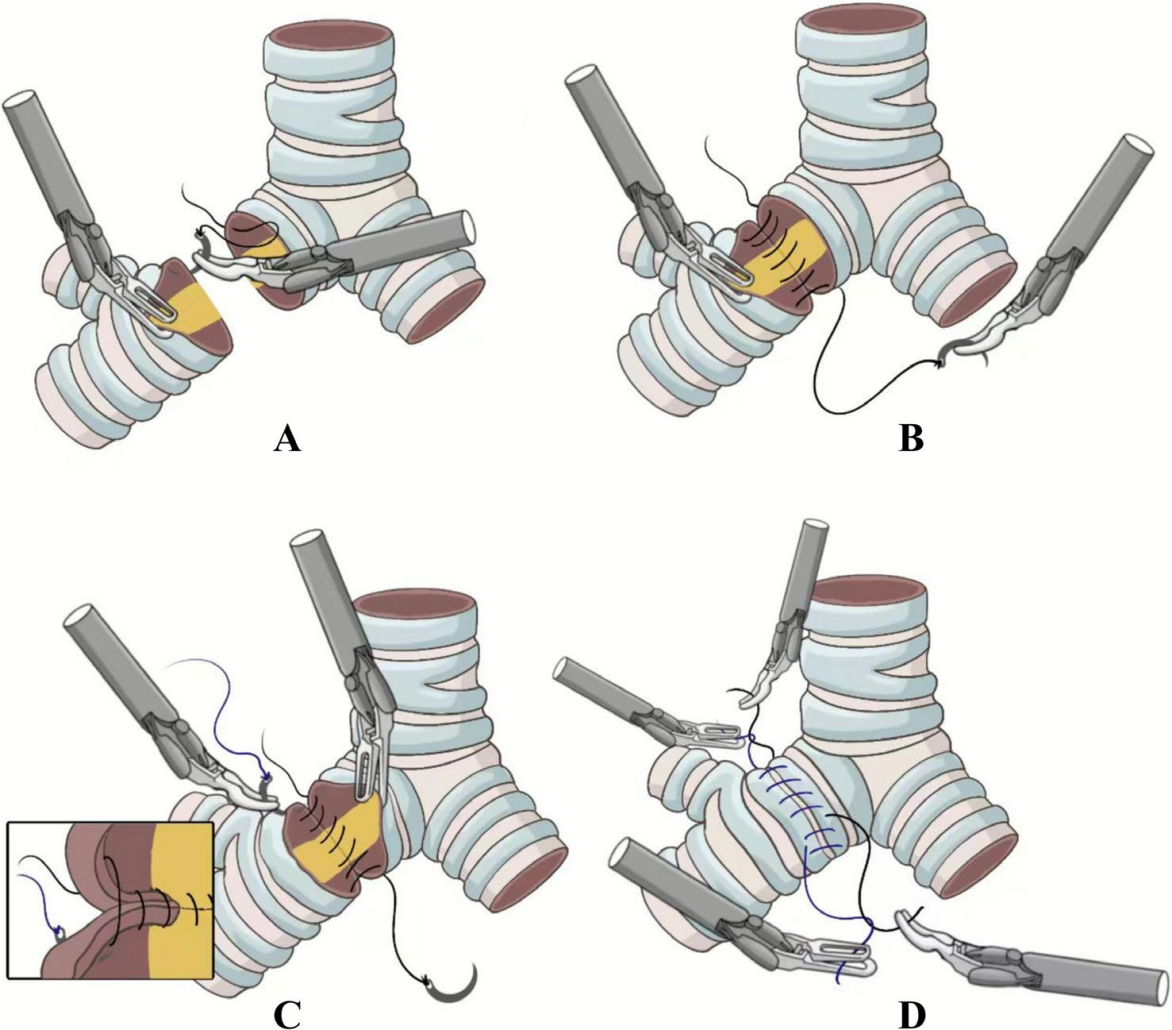
Semi‐continuous suture group (SCS): The bronchial anastomosis was completed using a double‐armed 3–0 Prolene suture in a semi‐continuous technique. (A) Initial needle entry at the lateral membranous‐cartilaginous junction. The needle penetrates the endoluminal surface of the contralateral cartilaginous stump and exits laterally. (B) “Extraluminal‐to‐extraluminal” continuous suturing along the membranous portion. Suturing terminates at the contralateral cartilaginous ring; tightened bidirectionally. (C) Second‐suture closure of the cartilaginous portion. A new suture repeats the “extraluminal‐to‐extraluminal” pattern from the initial entry point. (D) Independent ligation of suture origins and termination points. Separate knotting at entry/exit sites completes the anastomosis.

Using 3–0 Prolene sutures, the semi‐continuous technique was employed for anastomosing the proximal bronchus to the distal lobar bronchus: ① Commencing at the lateral aspect of the membranous‐cartilaginous junction, the needle was introduced through the endoluminal surface of the cartilaginous portion on the contralateral stump and exited laterally.② Subsequent suturing followed an “extraluminal‐to‐extraluminal” continuous pattern along this trajectory, terminating at the lateral aspect of the contralateral cartilaginous ring (opposite the starting point). The suture was tightened bidirectionally to achieve airtight approximation. ③ A second suture originated at the initial entry point, repeating the “extraluminal‐to‐extraluminal” continuous pattern to complete the anterior wall circumferential closure. Bidirectional tensioning was reapplied. ④ The suture origins (entry points) of both sutures were ligated and trimmed. The termination points (exit sites) were subsequently ligated and cut, completing the anastomosis (Figure 3).

### 2.5 Statistical Analysis

For continuous variables, if normally distributed, independent samples *t* tests were used for intergroup comparisons, with results expressed as mean ± standard deviation (x̄ ± s). For nonnormally distributed data, nonparametric tests (Mann–Whitney U tests) were employed, with results reported as median (interquartile range) (M [IQR]). Categorical variables were analyzed using chi‐square tests or Fisher's exact tests, with results presented as number (percentage) (*n* [%]). All statistical analyses were performed using SPSS 26.0 (IBM), with two‐tailed tests adopted and *p* < 0.05 considered statistically significant.

## 3 Results

This study enrolled 32 patients undergoing robot‐assisted bronchial sleeve anastomosis (SCS group: *n* = 18; CS group: *n* = 14). The two groups showed balanced baseline characteristics (all *p* > 0.05), including age (57.2 ± 11.2 years vs. 60.3 ± 10.8 years), sex (male 61.1% vs. 64.3%), BMI (25.3 ± 2.1 vs. 24.8 ± 3.5 kg/m^2^), and proportion of stage III tumors (72.2% vs. 64.3%). The SCS group demonstrated a significantly shorter anastomotic time (median 28 [24–33] min vs. 45 [32–52] min, *p* < 0.001) and a 21‐min reduction in operation time (135 [110–185] min vs. 156 [138–212] min, *p* = 0.040), with no differences in lymph node dissection count or R0 resection rate (both *p* > 0.05) (Tables 1 and 2).

**TABLE 1:** Preoperative patient characteristics.

Parameter	SCS (*n* = 18)	CS (*n* = 14)	*p*
Age, years	57.2 ± 11.2	60.3 ± 10.8	0.434
Sex			0.854
Male	11 (61.1%)	9 (64.3%)	
Female	7 (38.9%)	5 (35.7%)	
BMI, kg/m^**2**^	25.3 ± 2.1	24.8 ± 3.5	0.642
Smoking history			0.688
Yes	9 (50.0%)	8 (57.1%)	
No	9 (50.0%)	6 (42.9%)	
Hypertension			0.341
Yes	6 (33.3%)	7 (50.0%)	
No	12 (66.7%)	7 (50.0%)	
Diabetes mellitus			0.305
Yes	7 (38.9%)	8 (57.1%)	
No	11 (61.1%)	6 (42.9%)	
Neoadjuvant therapy			0.960
Yes	13 (72.2%)	10 (71.4%)	
No	5 (27.8%)	4 (28.6%)	
Preoperative lung function			
FEV1 (% predicted)	90.0 ± 12.0	92.0 ± 17.0	0.712
DLCO (% predicted)	88.0 ± 16.0	91.0 ± 14.0	0.576
Tumor stage			0.507
Stage I	1 (5.6%)	0 (0.0%)	
Stage II	4 (22.2%)	5 (35.7%)	
Stage III	13 (72.2%)	9 (64.3%)	
Histological type			0.376
Squamous cell carcinoma	11 (61.1%)	9 (64.3%)	
Adenocarcinoma	5 (27.8%)	3 (21.4%)	
Other	2 (11.1%)	2 (14.3%)	

*Note:* Continuous data are expressed as mean ± SD; categorical data as *n* (%).

Abbreviations: %pred, percentage predicted; DLCO, diffusing capacity of the lung for carbon monoxide; FEV1, forced expiratory volume in 1 s.

**TABLE 2:** Intraoperative data.

Parameter	SCS (*n* = 18)	CS (*n* = 14)	*p*
Sleeve resection location			0.652
Right upper lobe	8	5	
Right middle/lower lobe	2	3	
Right lower lobe	3	2	
Left upper lobe	3	3	
Left lower lobe	2	1	
Anastomosis time, min	28.0 [24.0–33.0]	45.0 [32.0–52.0]	< 0.001
Operative time, min	135.0 [110.0–185.0]	156.0 [138.0–212.0]	0.040
Number of lymph nodes dissected	16.2 ± 1.8	17.3 ± 1.7	0.096
R0 resection margin status	18	14	0.999

Postoperative safety outcomes revealed comparable overall anastomosis‐specific complication rates (11.1% vs. 21.4%, *p* = 0.425), including similar incidences of atelectasis and bronchopleural fistula (both *p* > 0.05); no significant differences in overall nonspecific complication rates (22.2% vs. 42.9%, *p* = 0.212) or individual events (empyema 5.6% vs. 14.3%, *p* = 0.401; postoperative hemorrhage 0% vs. 7.1%, *p* = 0.249). The groups showed 90‐day mortality (0% vs. 7.1%), late stenosis rate (0% vs. 7.1%), and reoperation rate (5.6% vs. 14.3%) (all *p* > 0.05), with no significant differences in extubation time (4.09 ± 0.94 days vs. 4.56 ± 0.67 days, *p* = 0.109) or postoperative hospital stay (6.97 ± 1.26 days vs. 7.26 ± 1.59 days, *p* = 0.581). (Table 3).

**TABLE 3:** Postoperative outcomes.

Parameter	SCS (*n* = 18)	CS (*n* = 14)	*p*
Transfer to ICU			0.928
Yes	8	6	
No	10	8	
Time to extubation, days	4.09 ± 0.94	4.56 ± 0.67	0.109
Postoperative hospital stay, days	6.97 ± 1.26	7.26 ± 1.59	0.581
Specific early complications	2	3	0.425
Atelectasis	1	1	0.854
Bronchial fistula	1	2	0.401
Nonspecific early complications	4	6	0.212
Pneumonia	3	2	0.854
Postoperative hemorrhage	0	1	0.249
Empyema	1	2	0.401
90‐day mortality	0	1	0.249
Late stenosis (> 90 days)	0	1	0.249
Reoperation rate	1	2	0.401

## 4 Comment

This study represents the first systematic comparison of CS versus SCS techniques in RATS sleeve lobectomy, establishing their respective clinical merits. The key finding demonstrates that the SCS technique safely reduces the bronchial anastomotic time during RATS sleeve resections. This conclusion provides critical evidence to optimize robotic bronchial reconstruction.

From a technical perspective, in traditional CS, when using a sliding suture for anastomosis, it is necessary to work with an assistant to pull the suture every four to six stitches to maintain tension and prevent the suture from loosening [13]. This process can easily lead to suture entanglement or breakage, which undoubtedly prolongs the surgical procedure. In contrast, SCS employs a two‐segment 10 cm 3–0 Prolene suture for stepwise suturing: this allows the surgeon to manage local tension independently without assistant intervention, significantly reducing operational conflicts of robotic long‐arm instruments within the narrow thoracic cavity. Crucially, SCS only requires ensuring physiological alignment of the bronchial resection margin, avoiding the cognitive load associated with the complex three‐dimensional structure at the membrane‐cartilage junction in traditional CS techniques. Data from this study show that the anastomosis time is reduced in the SCS group, with its efficiency advantage directly stemming from reduced suture management actions and the parallelisation of membrane/cartilage segment operations.

For patients with locally advanced central‐type lung cancer undergoing neoadjuvant immunochemotherapy, sleeve resection is often required to ensure negative surgical margins [14]. In this study, 72.2% of patients in the SCS group received neoadjuvant therapy, and the incidence of anastomotic fistula remained comparable to that in the CS group (5.6% vs. 14.3%, *p* = 0.401), confirming the oncological applicability of SCS technology. Anastomotic stenosis is a key factor affecting postoperative recovery and prognosis [15]. The segmented knot‐tying mechanism avoids the “purse‐string effect” caused by continuous single‐line traction, thereby reducing the risk of long‐term stenosis from a mechanical perspective. Previous studies have suggested that CS may increase the risk of stenosis [16], but no such difference was observed in this study, potentially due to the unique advantages of robotic technology. The robotic system's × 10 magnification of the surgical field ensures precise control of stitch spacing and margin distance, thereby preserving the integrity of bronchial microcirculation.

However, this study still has limitations. As a single‐center retrospective study, the sample size is relatively small and subject to selection bias. This study was performed by a single team with a standardized protocol. Nevertheless, the specific practices and patient baseline characteristics inherent to this setting might restrict the broader applicability of our conclusions. Future studies should include multicenter, large‐sample prospective randomized controlled trials to further validate the efficacy of the SCS technique in different healthcare settings. Additionally, long‐term outcomes, such as long‐term pulmonary function recovery, should be monitored to provide more comprehensive evidence‐based support for widespread clinical application.

SCS technology uses double‐line segmented suturing and an autonomous tension adjustment mechanism to significantly improve the efficiency of RATS bronchial anastomosis without increasing the risk of complications, while avoiding reliance on assistants and simplifying thread management. It is suitable for complex anatomical reconstruction following neoadjuvant therapy, providing a safe, efficient, and universally applicable technical option for robotic sleeve resection.

## Author Contributions

Conception and design: Hao Zhang and Yong Ge. Administrative support: Hao Zhang. Provision of study materials or patients: Zhiqiao Chen, Shuyuan Li, and Yongxin Fan. Collection and assembly of data: Xinyu Zhu, Shoujie Feng, Qi Tang, and Xuanyi Zong. Data analysis and interpretation: Teng Sun and Cheng Zhang. Manuscript writing: All authors. Final approval of manuscript: All authors.

## Funding

This work was supported by the Noncommunicable Chronic Diseases‐National Science and Technology Major Project (2024ZD0529400 & 2024ZD0529405), The National Natural Science Foundation of China (82472885), Xuzhou “Pengcheng Talent Program”‐High‐level Healthcare Talent Recruitment and Development Project (2025DF04 & 2025DJ02), The Social Development Projects of Key R&D Programs in Xuzhou City (KC22097), and the Xuzhou Medical University 2022 Scientific Research Launch Fund for Introducing High level Talents (D2022016).

## Ethics Statement

The study was conducted in accordance with the Declaration of Helsinki and approved by the ethics committee of the Affiliated Hospital of Xuzhou Medical University. Due to the retrospective nature of the study, the requirement for informed consent was waived.

## Conflicts of Interest

The authors declare no conflicts of interest.

## Data Availability

The data that support the findings of this study are available from the corresponding author upon reasonable request.
